# Natural cycles in South Pacific Gyre strength and the Southern Annular Mode

**DOI:** 10.1038/s41598-022-22184-2

**Published:** 2022-10-27

**Authors:** Nicholas T. Hitt, Daniel J. Sinclair, Helen L. Neil, Stewart J. Fallon, Aimée Komugabe-Dixson, Denise Fernandez, Philip J. Sutton, John C. Hellstrom

**Affiliations:** 1 Victoria University of Wellington, Wellington, New Zealand; 2 National Institute of Water and Atmospheric Research, Wellington, New Zealand; 3 Australian National University, Canberra, Australia; 4 Global Fishing Watch, Washington, DC USA; 5 University of Melbourne, Melbourne, Australia

**Keywords:** Climate sciences, Palaeoceanography, Palaeoclimate

## Abstract

The South Pacific Gyre (SPG) plays a vital role in regulating Southern Hemisphere climate and ecosystems. The SPG has been intensifying since the twentieth century due to changes in large scale wind forcing. These changes result from variability in the Southern Annular Mode (SAM), causing warming along the eastern SPG which affects local ecosystems. However, our understanding of SPG variability on timescales greater than several decades is poor due to limited observations. Marine sediment cores are traditionally used to determine if recent ocean trends are anomalous, but rarely capture centennial variability in the southwest Pacific and limit our understanding of SPG variability. Here we capture centennial SPG dynamics using a novel high-resolution paleocirculation archive: radiocarbon reservoir ages (R) and local reservoir corrections (∆R) in SPG deep-sea black corals. We find black coral R and ∆R correlates with SAM reconstructions over 0–1000 cal BP and 2000–3000 cal BP. We propose this correlation indicates varying transport of well-ventilated subtropical waters resulting from SPG and SAM interactions. We reconstruct several ‘spin up’ cycles reminiscent of the recent gyre intensification, which has been attributed to anthropogenic causes. This implies gyre strength and SAM show natural co-variability on anthropogenic timescales which should factor into future climate projections.

## Introduction

The South Pacific Gyre (SPG) results from an interaction between atmospheric winds and surface waters^1–17^. Basin-wide atmospheric wind patterns in the tropical and subtropical Pacific Ocean are driven by a combination of the Hadley and Walker Circulation patterns and the Coriolis force^2,15^. These atmospheric features create a pattern of easterly winds near the equator (the trade winds) and westerly winds at the mid-latitudes^15–17^, which drive a series of a surface anticyclonic ocean currents that distribute temperature and nutrients across the Pacific basin^2^. This intricate interplay is modulated by natural climate variability in the South Pacific through complex air-sea interactions. This includes the Southern Annular Mode (SAM)^8,18^, which is the leading mode of climate variability in the southern mid-latitudes and has undergone significant change over the twentieth century^19^.

**Figure 1:**
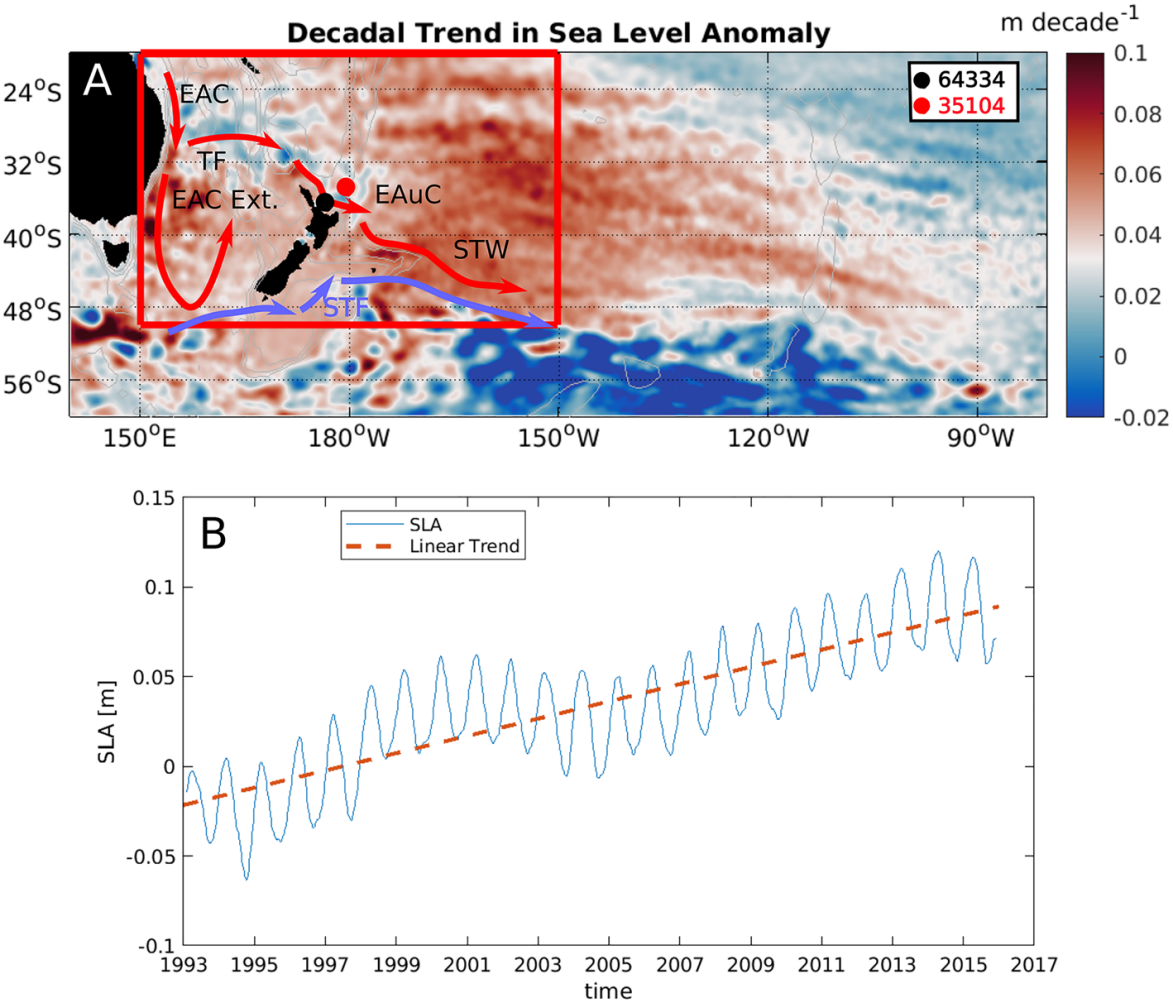
Regional Trends in Southwest Pacific Gyre Oceanography. (**A**) The trend in South Pacific Gyre sea level height anomalies (SLA) for the 1993-2016CE period. Trends are calculated using weekly values of sea level height anomalies (SLA). Data are from AVISO Ssalto/Duacs altimeter products, the delayed time multi-mission project produced and distributed by the Copernicus Marine and Environment Monitoring Service (CMEMS) (http://www.marine.copernicus.eu). Red areas indicate a positive SLA trend, blue areas indicate a negative SLA trend. Red arrows indicate warm subtropical tropical currents and blue arrows indicate cool subtropical currents; EAC—East Australian Current; TF—Tasman Front; EAC Ext.—East Australian Current Extension; EAuC—East Auckland Current; STW – Subtropical Water; STF—Subtropical Front. Colored dots indicate the coral locations in the study. (**B**) The trend in Southwest Pacific Gyre Sea Level Height Anomalies for the 1993–2016CE period. The trend (red line) is calculated using a linear fit for each grid point and then constructing a spatial average over the selected region (red box in Panel **A**) and values are plotted as function of time (blue line). Seasonality is not removed.

The SAM modulates the position and strength of the westerly wind belt, which assists in driving the equator to pole heat flux via the strength and position of wind-driven western Pacific boundary currents^18^. Over the late twentieth century, the SAM has been trending towards a more positive state, consistent with more intense westerlies^19^. This has increased the flux of warmer waters into the Southwest Pacific (Fig. 1)^3,5,9,20,21^, leading to increased ocean stratification, an increase in sea level height, and warmer surface ocean temperatures. These changes have altered the oceanographic and biological landscape of the southwest SPG, contributing towards frequent marine heat waves and a reduction in primary productivity^22,23^ which threaten native fisheries that support a $2 billion local economy^6,10,24,25^.

Although these changes appear to be significant in the context of recent decades, oceanographic observations are sparse in space and time prior to the satellite era beginning in 1979^11,26^. The lack of in situ data creates considerable uncertainty in ascertaining the significance of recent ocean changes in the context of natural climate variability and anthropogenic forcing^11^. Understanding the natural cycles in recent ocean changes therefore requires additional records where a baseline pattern of natural ocean variability can be used as a reference and contextualize recent ocean change. Unfortunately, traditional proxy records like marine sediment cores and tropical corals are either geographically restricted or lack sufficient resolution to assess low-frequency variability over the twentieth century in the mid-latitude southwest Pacific^12,13,27^. This represents a significant barrier to developing and improving our understanding of mid-latitude southwest Pacific oceanography.

These knowledge gaps can be filled using deep-sea black corals^14^. Black corals have a branching organic skeleton with a thick basal trunk that displays growth rings much like a tree, making it straightforward to establish growth direction. Moreover, black corals can also live for millennia with growth rates suitable for extracting information with decadal to sub-decadal resolution^14,28^. Therefore, black corals offer a new opportunity to advance our understanding of natural centennial to millennial ocean changes and how those changes pertain to anthropogenic climate change^14,29–31^.

Black coral skeletons are built from, and thus inherit, the isotopic signature of the organic material that the corals consume: primarily detrital plankton matter originating in the surface ocean^14,28^. This includes radiocarbon (^14^C), whose concentration varies spatially in phytoplankton due to fluctuating ^14^C concentrations in different ocean current regimes (Supplementary Fig. 1)^32–35^. Radiocarbon in black corals has been used to study ocean circulation by calculating sea surface radiocarbon reservoir ages (R) and local surface ocean reservoir correction factors (ΔR)^14,36^. R is defined as the difference between the ^14^C age of the sea surface and atmosphere due to the lag caused by air-sea exchange of CO_2_ and dilution through oceanic mixing and circulation^14,36^. ∆R is the difference between the measured ^14^C age of the surface ocean and a ^14^C age predicted from the global marine radiocarbon curve ‘Marine20’^37,38^. R and ∆R track the balance of ‘old’ subsurface waters and ‘young’ surface waters, and indicate where local processes (e.g. circulation, upwelling) shift the surface water’s age away from that of the modelled average ocean or atmosphere^14,35–38^. R and ∆R are typically high in old subsurface waters due to radioactive decay of ^14^C, and low in surface water due to consistent air-sea gas exchange.

R and ∆R are reconstructed by pairing measured radiocarbon age with an age constraint by an independent dating technique, such as uranium-thorium (U-Th) disequilibrium ages or a known calendar age^14,37^. Calculating R and ∆R from black corals has previously been done by pairing radiocarbon (^14^C) and U-Th ages using the following equations^37,62^:

$${\text{R }} = {\text{ measured}}\,^{{{14}}} {\text{C}}\,{\text{ age }}{-}{\text{ SHCal2}}0^{{{61}}} \,{\text{modelled}}\,^{{{14}}} {\text{C}}\,{\text{ age}}$$

$$\Delta {\text{R }} = {\text{ measured}}\,^{{{14}}} {\text{C}}\,{\text{ age }}{-}{\text{ Marine2}}0\,{\text{ modelled}}\,^{{{14}}} {\text{C}}\,{\text{ age}}$$

Black corals are particularly well suited to reconstructing R and ∆R since their skeletons are rich in uranium (~ 20 ppm), can be precisely dated by U-Th^14,36^, and capture the ^14^C age of the surface exported organic matter they feed on.

Here we use R and ∆R in corals collected from the East Auckland Current (EAuC) north of Aotearoa New Zealand to reconstruct baseline SPG circulation variability over the late Holocene (Fig. 1A; Suppementary Table 1). These records effectively reconstruct western SPG boundary current strength and the intensity of SPG circulation.

## Results

**Figure 2:**
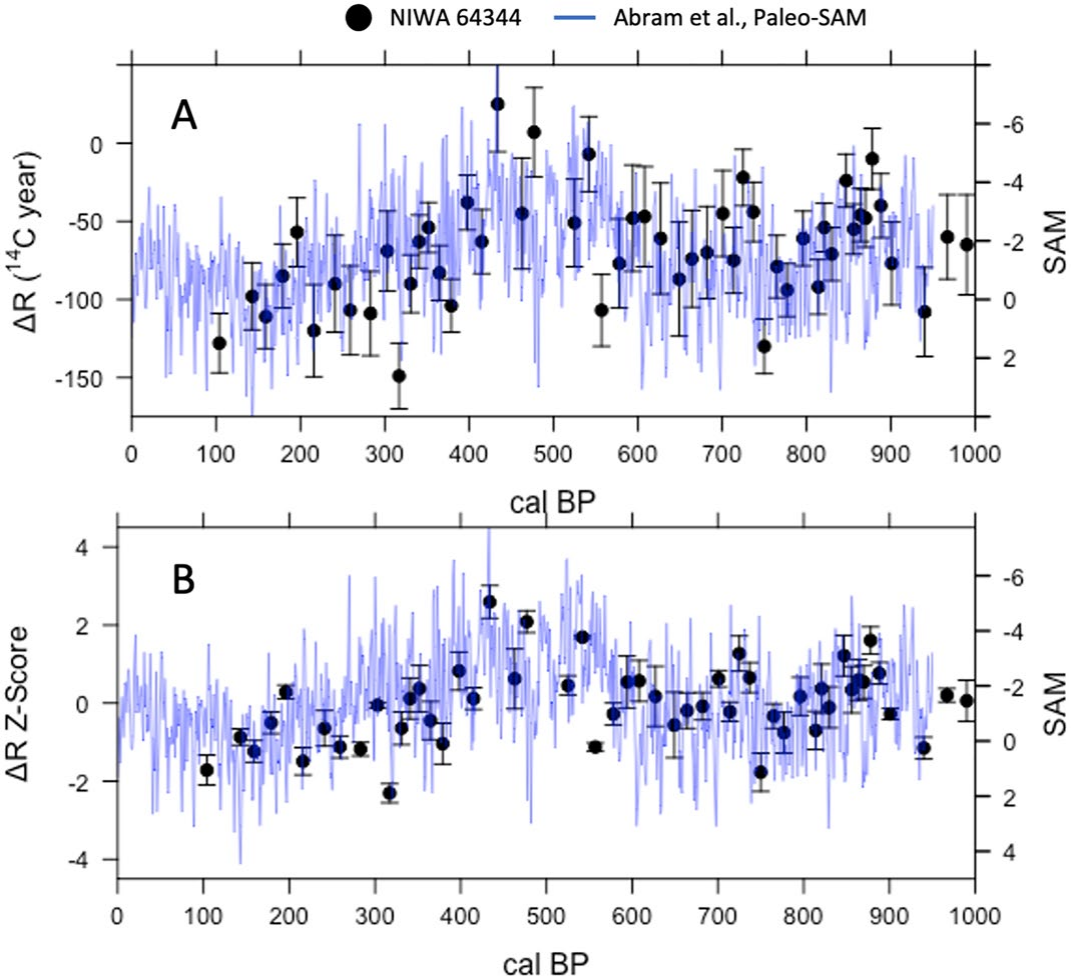
Subtropical SPG ∆R Variability and Paleo-SAM over the last Millennium. (**A**) A comparison of black coral ∆R values for the East Auckland Current (black dots—NIWA 64344) with the paleo-SAM index from Abram et al.^39^ (blue line). (**B**) A comparison of ∆R normalized Z-scores (black dots) against Paleo-SAM (blue line). All uncertainties shown are 1σ, are calculated using the Reimer et al.^37^ methodology, and reflect the uncertainty in the radiocarbon age and U-Th calendar age.

**Figure 3:**
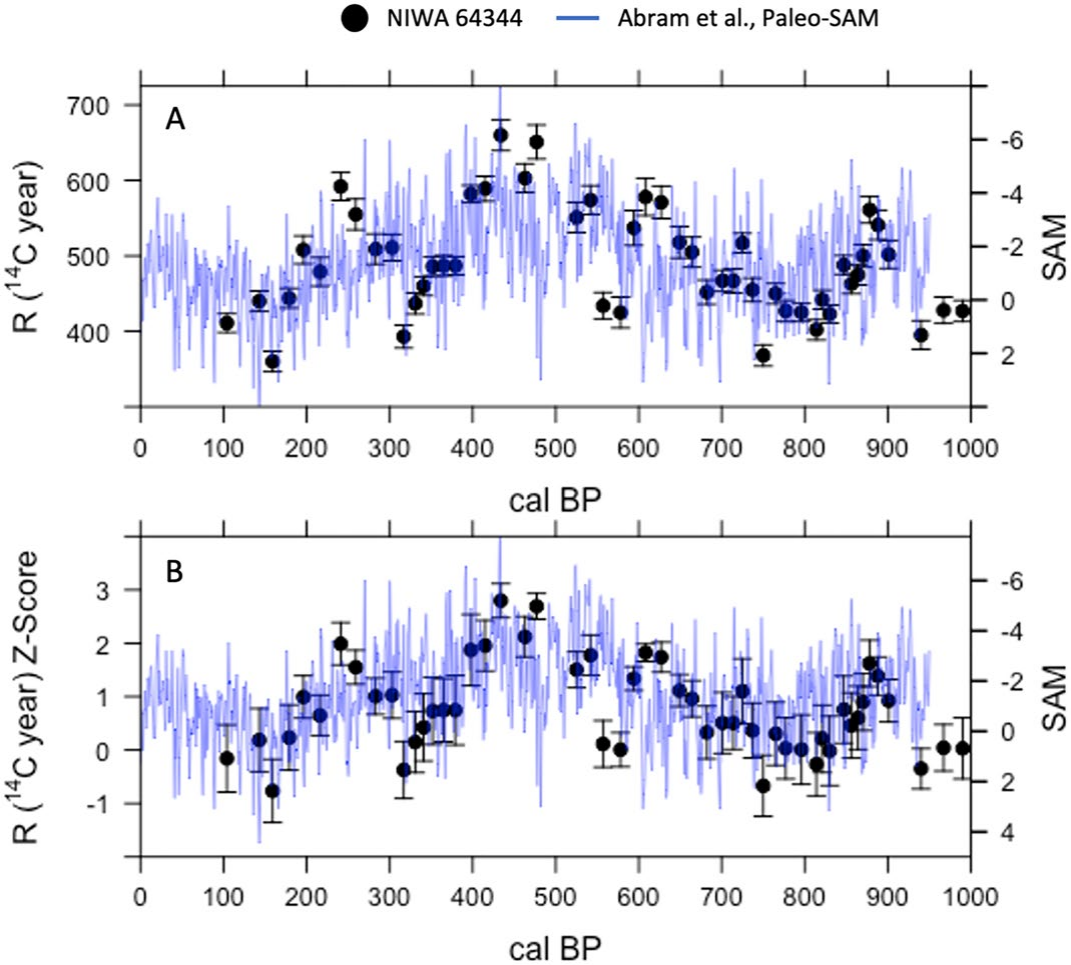
Subtropical SPG ∆R Variability and Paleo-SAM over the last Millennium. (**A**) A comparison of black coral ∆R values for the East Auckland Current (black dots—NIWA 64344) with the paleo-SAM index from Abram et al.^39^ (blue line). (**B**) A comparison of ∆R normalized Z-scores (black dots) against Paleo-SAM (blue line). All uncertainties shown are 1σ, are calculated using the Soulet^62^ methodology, and reflect the uncertainty in the radiocarbon age, U-Th calendar age, and the SHCal20 curve^61^.

Black coral R and ΔR from the EAuC show variability of up to 250 ^14^C years (R) and 150 ^14^C (∆R) years on multi-centennial timescales, respectively (Figs. 2A, 3A). These fluctuations imply some periodic change in R and ∆R in the East Auckland Current which may be indicative of natural ocean variability. Previous work in the southwest Pacific showed R and ∆R variations represented variations in gyre strength which changed the flux of tropical-sourced waters with lower R and ΔR values into the southwest Pacific^14,36^. Variations in R and ∆R are synchronous and have a strong linear relationship and positive correlation (m = 0.80; r = 0.90, *p* < 0.0001; Supplementary Fig. 10).

In modern times, the strength of western SPG currents has been associated with variations in the mean state of the SAM^3,8,21^. We test whether SAM may have also acted as a climatic driver of R and ∆R variability in the late Holocene by comparing our records with the paleo-SAM index from the last millennium generated by Abram et al.^39^ (Figs. 2, 3). This index is constructed from a network of Antarctic ice cores and South American tree rings and represents sea level pressure anomalies between 40 and 60°S across Drake Passage. Although there are other climate modes at play in that region which interact with the SAM (including the Zonal Wave 3 and Pacific South America Pattern^41,42^), the Abram et al.^39^ Paleo-SAM index is regarded as the best paleoclimate record of SAM currently available^43^.

**Figure 4:**
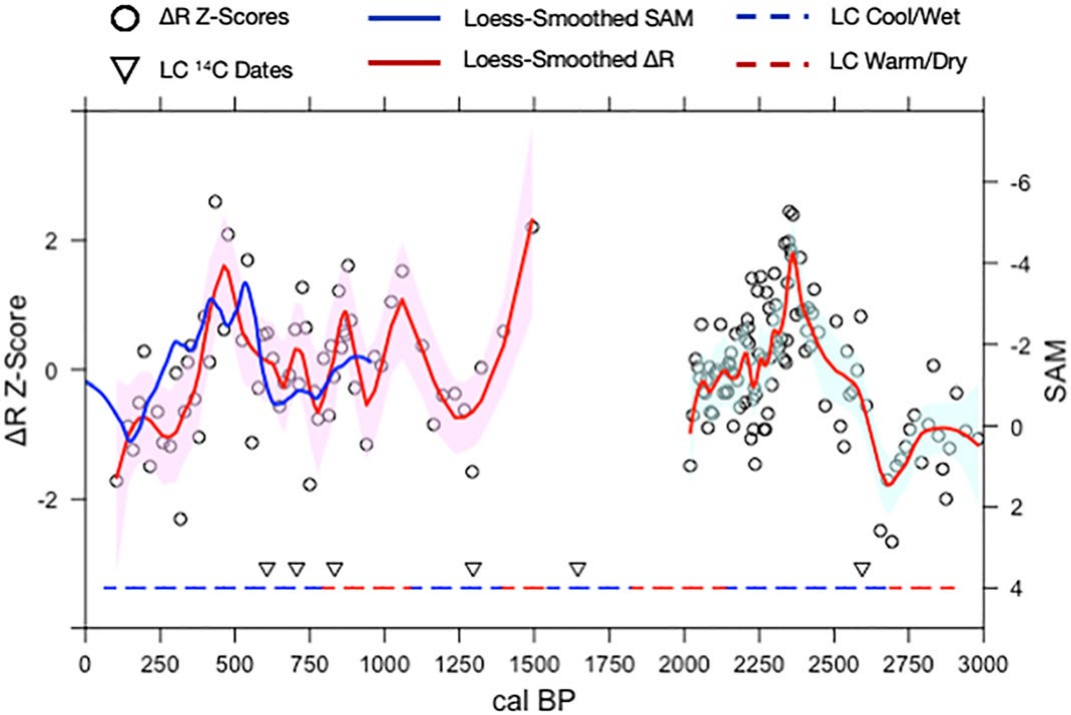
Subtropical SPG ∆R Variability and Paleo-SAM over the Late Holocene. Loess filtered proxy data (Abram et al.^39^ Paleo-SAM—blue; Z-Score normalized ∆R—red) overlaid on top of Z-Score normalized ∆R data (black open circles). Loess filtering uses a λ of 0.2. Light red shading shows a confidence interval of 95%. ∆R data older than 950 cal BP is used as a proxy record to infer SAM conditions over the late Holocene. Red and blue dashed lines represent the inferred cool/wet and warm/dry periods from Ciprese Cycles (CC#) in Patagonia, South America^40^ complete with radiocarbon dates for the LCNAP record (inverted white triangles with black outline). The and the correlation between loess-filtered paleo-SAM and a z-score normalization of the 64344 ∆R record is r = − 0.54 (*p* < 0.01; n = 50).

We compare the EAuC R and ∆R records (NIWA 64344) with the Abram et al.^39^ SAM index using a correlation and regression analysis to better understand the relationship between SPG circulation and the SAM over the last millennium (Figs. 2, 3). We first normalize the EAuC R and ∆R records to z-scores (μ of 0 and σ of 1) since the R, ∆R, and paleo-SAM records exist on different scales (Fig. 2B, 3B). We then fit a local polynomial regression (a.k.a. loess-filter) with a λ = 0.2 to each record to identify multi-centennial trends (Fig. 4). This method leverages all the data in each record to produce evenly spaced records with lower noise and highlights common variations without significantly muting the intrinsic variability in each record. We quantitatively assess the relationships between R and ∆R and SAM using a Pearson’s and Spearmen’s correlation and a regression analysis^45^ on the evenly spaced loess-filtered records (Supplementary Figs. 7, 8).

The z-score normalized EAuC R and ∆R records (NIWA 64,344) correlate well with the Abram et al.^39^ SAM index (Fig. 2B, 3B and 4; Supplementary Figs. 7, 8), with persistently high R and ΔR z-scores occurring during multi-centennial periods of negative SAM. A bootstrapped (n = 10,000) Pearson’s and Spearmen rank order correlation on loess-filtered R and ΔR z-scores and SAM data shows a moderate inverse correlation over the last millennium (r = − 0.49 to − 0.55, *p* < 0.001; n = 50; Fig. 4) and a linear regression confirms a strong and statistically significant relationship where the records overlap (Supplementary Figs. 7, 8).

This negative correlation accords with our understanding of modern SAM/SPG dynamics. During positive SAM phases, the westerly wind belt shifts south and strengthens, transporting more warm subtropical water into the southwest Pacific^3,8,18,21^. These warm subtropical waters are more ventilated to the atmosphere and have lower R and ΔR values^35,44^, which produces the negative relationship. This air-sea interaction has been observed since the late 1980s, and our reconstructions imply a similar interplay between SAM and SPG circulation has likely been a feature of South Pacific climate over the last millennium. However, a limiting feature for comparing the two proxy records is the length of the Abram et al.^39^ Paleo-SAM record, which only dates back to 950 cal BP. Ensuring multi-centennial variability in southwest Pacific black coral radiocarbon beyond 950 cal BP reflects changes in gyre circulation which is driven by paleo-SAM variability (and may therefore serve as a proxy record of paleo-SAM) requires a comparison with additional paleo-SAM proxy records beyond 950 cal BP. We base our discussion on the relationship between gyre circulation and paleo-SAM variability beyond 950 cal BP on ∆R values for the sake of brevity  since R and ∆R are positively related and show similar synchronous variability (Figs. 2, 3; Supplementary Fig. 10), and because R, ∆R, and the residual difference between black coral ∆^14^C and the ∆^14^C of SHCal20 are all correlated with each other as well as the paleo-SAM index over 950 to 0 cal BP (Supplemental Text; Supplementary Figs. 9, 10 and 11).

We use loess-filtered ∆R z-scores across the 1500–950 cal BP section of NIWA 64344 and 2982–2019 cal BP section of NIWA 35104 as a proxy record of paleo-SAM variability over the late Holocene. We note the omission of the 2019 to 578 cal BP section of the NIWA 35104 record in our analysis and figures. This is because over the 2019 to 578 cal BP interval NIWA 35104 exhibits a rapid change to unprecedented slow coral growth (~ 1 µm/year)^28^ and large uncertainties in ∆R calculations due to high initial ^230/232^Th values (54 ± 11). In this interval, the U-Th ages used for ∆R calculations become sparser and require intense interpolation to match the sampling resolution of ^14^C dates, making ∆R values statistically indistinguishable (Supplementary Fig. 5). The uncertainty is compounded by elevated detrital Th which is notably higher than the older portion of this coral, and other corals in the Southwest Pacific (see “Methods” section)^14^. It is possible the slow growth prohibited a closed-system behavior required for accurate U-Th aging techniques. Interpreting ∆R data from this section of the coral as climatologically forced or representative of water mass dynamics therefore carries significant risk. We therefore remove the 2019 to 574 cal BP section of 35104 from our analysis but provide all data in the supplementary material and data.

We validate loess-filtered ∆R z-scores as a proxy record of paleo-SAM variability beyond 950 cal BP using a reconstruction of eastern South Pacific basin non-arboreal pollen (NAP) records from Lago Ciprese in Patagonia (LCNAP)^40^. The LCNAP record captures so-called ‘Ciprese Cycles’ (CC) which represent periods of warm/dry conditions that serve as analogues for positive SAM-type states in Patagonia^40^. We observe a good correspondence between our loess-filtered ∆R z-scores and CCs over the 3000 to 2000 cal BP period. CC5 precisely matches up with our most negative loess-filtered ∆R z-scores over the last 3000 years, highlighting a period of more positive SAM conditions and stronger gyre circulation (Fig. 2B). We note that there is some uncertainty in the onset and timing of CC5 which may result from low-resolution radiocarbon dating constraints in the LCNAP record and/or the use of different prior versions of the calibration curves (IntCal) used to construct the LC NAP age-depth model (Fig. 4; Supplemental Data Table 4)^40,46^. Nonetheless, the inferred shifts in SAM states between the CC from the LC NAP record and the loess-filtered ∆R z-scores agree well over the 3000 to 2000 cal BP period, although, this relationship appears to be non-stationary over the Late Holocene.

In contrast to the consistent patterns of variation seen between 3000 and 2000 cal BP, the relationship is less clear over the 1500 to 950 cal BP interval due to some disagreement between the records. Our loess-filtered ∆R z-scores become inversely correlated with the LC NAP record between 1395 to 1086 cal BP (Fig. 4). The LC NAP record is also contradicted by the Abram et al.^39^ SAM index and ∆R reconstruction between 950 to 794 cal BP. Although there is some corroborating evidence supporting the LC NAP record around the 1100 to 900 cal BP interval^47^, the exact state of paleo-SAM between 1500 and 900 cal BP remains unclear. At present it is not clear which of the records faithfully tracks SAM over this interval. An interesting possibility is that the disagreement arises from a zonally-asymmetric SAM pattern over the 1500 to 900 cal BP interval, which may decouple the loess-filtered ∆R z-scores from the LC NAP record. It is also possible that some of, or none of the variability seen in the various proxy records (LC NAP, Abram et al.^39^, ∆R) reflects paleo-SAM. It is also possible that ∆R is decoupled from SAM over 1500–900 cal BP interval. However, each of these possibilities are purely speculative and the exact cause for the discrepancy between the ∆R record and the paleo-SAM reconstructions over 1500–900 cal BP cannot be determined with the present information.

## Conclusion

We conclude that the paleo-SAM conditions inferred from our ∆R records are robust over the last millennium and 3000 to 2000 cal BP period based on the relative agreement between our ∆R reconstructions, the Abram et al.^39^ Paleo-SAM index, and the inferred SAM state from the Lago Cipreses pollen record. We propose the ΔR-SAM relationship arises from coupled SPG-SAM circulation. Our ∆R reconstructions suggest the late Holocene experienced multi-centennial-long periods of enhanced gyre strength and is forced by SAM conditions.

Our records imply the recent increase in SPG circulation strength may be a persistent feature of the late Holocene, and modern changes to the SPG are not unprecedented over the last few millennia. Clearly this system has significant internal variability on the order of centuries. These natural cycles in gyre strength should be considered in future projections of ocean change in the southwest Pacific and used as a baseline when debating an anthropogenic cause or identifying anthropogenically driven trends in coupled ocean-atmosphere changes^21,48,49^.

The influence of SAM on the SPG likely impacts the biological oceanography of the southwest Pacific^8,18^. The warm subtropical waters advected into the southwest Pacific by positive SAM phases are macro-nutrient depleted^2,8,18,21^, and warmer waters increase vertical ocean stratification. Both factors tend to reduce nutrient supply to the surface ocean and therefore suppress primary productivity^2,9,10,23^. This has been observed in chlorophyll satellite data^6,26^, which further highlights the need to investigate connections between SPG air-sea interactions and biology. Our reconstructions have identified several periods that could serve as modern analogues for these efforts, which could leverage existing deep-sea coral paleocean archives^30,31,50^.

Improving future projections of change to this region requires a baseline point of reference natural variability to quantify the influence of anthropogenic change. Our study, like others^21,39,40,49^, is not able to fully constrain the external climate forcings driving coupled variations in SPG and SAM due to limits in record availability; however, we show this barrier can be overcome using radiocarbon isotopes stored in black coral skeletons. Such efforts will improve knowledge of baseline ocean variability and assist in contextualizing recent anthropogenically-driven ocean changes.

## Methods

### Coral sample description, preparation and radiocarbon dating

Two New Zealand black coral colonies (species *Leiopathes *spp*.*) collected north of Aotearoa New Zealand were provided by the National Institute of Water and Atmospheric Research Invertebrate Collection in Wellington, New Zealand (Supplemental Table 1; Fig. 1A). Powdered samples were generated by milling whole rings using a MicroMill DSLS 3000 Desktop Machining System CNC milling machine (Chandler, Arizona, USA). Radiocarbon measurements were made on randomly selected, cleaned splits of the powders (~ 1 mg) on a single-stage Accelerated Mass Spectrometer (AMS) at the Australian National University Radiocarbon Laboratory following procedures outlined in Fallon et al.^51^. The coral radiocarbon results presented here are the same as those corals presented in Hitt et al.^28^. Full details of the sub-sampling and radiocarbon measurement protocols are presented in Hitt et al.^28^.

### U-Th dating

Previous studies show that black corals have a high U concentration (10–80 ppm) and exhibit closed system behavior which makes them ideal for U-Th dating^14,36^. U-Th ages were generated at 0.2–1 mm intervals across all corals (Supplementary Fig. 2, Supplemental Data Table 1).

U-Th analyses were conducted according to the procedure of Hellstrom^52^ modified according to Drysdale et al.^53^. Black coral sub-samples up to 2 mg were randomly selected and dissolved in ~ 15 M HNO_3_, spiked with a mixed ^236^U–^233^U–^229^Th synthetic isotopic tracer and equilibrated on a hotplate overnight at ~ 85 °C. Equilibrated samples were loaded onto 0.5 ml Eichrom TRU ion exchange columns and the black coral matrix washed off, prior to elution of U and Th as a single fraction. The purified U-Th samples were randomly analysed using a Nu Instruments ‘Plasma’ MC-ICP-MS at the University of Melbourne. Samples were introduced via a Nu Instruments DSN desolvator in 5% HNO_3_ and 0.5% HF using a Glass Expansion Opalmist Teflon nebulizer. All isotope ratios were determined simultaneously with the use of dual SEM ion counters for paired measurement of ^233^U/^234^U and ^229^Th/^230^Th. Most probable initial ^230^Th/^232^Th activity ratios were determined using the stratigraphic constraint technique of Hellstrom^54^, allowing calculation of corrected U-Th ages using the ^234^U and ^230^Th half-lives of Cheng et al.^55^. Th corrected U-Th dating results are provided in Supplementary Figs. 2, 3 and Supplemental Data Table 1.

### Calendar age versus depth model construction

Age-depth models were constructed from initial-Th corrected U-Th using COPRA^55^, a piecewise cubic interpolation spline with a Monte Carlo simulation (n > 2000; Supplementary Fig. 2, Supplemental Data Tables 2 and 3). It was necessary to interpolate U-Th ages in order to estimate the calendar age for each radiocarbon measurement because U-Th dates were generated at a lower frequency than ^14^C dates (~ 1 in 3).

After correction for initial-Th, coral 35104 displays U-Th age reversals, possibly due to the high density of U-Th dates. A total of 5 age reversals were seen over the 2000–3000 cal BP interval, each less than 30 years, and each occurring in intervals with a high density of surrounding U-Th dates (n > 3 in an interval of 125 years). Breitenbach et al.^56^ suggests that COPRA can construct an age model from a dataset with age reversals so long as the error intervals of these age reversals overlap. However, the overlapping age reversals occur in high density U-Th date intervals and prevent COPRA from constructing an age model in 35,104 (Sebastian Breitenbach, 2019, pers. comm.). We therefore removed the five age reversals over the 2000–3000 cal BP interval from the suite of U-Th ages (n = 30 to n = 25 over the 2000–3000 cal BP interval) used for age model construction as per the recommendation of the COPRA authors (Sebastian Breitenbach, 2019, pers. comm.). After removing age reversals, we still maintain the same U-Th date frequency observed in coral 64,344 (~ 1U-Th:3.5 ^14^C; 1U-Th:40–50 years).

### R, ΔR and Δ^14^C calculations

R values were calculated from interpolated U-Th ages and measured ^14^C ages using the *ResAge* R Package modified to include the SHCal20 calibration curve. Local marine radiocarbon reservoir correction factors (ΔR) were calculated from interpolated U-Th ages and measured ^14^C ages using the online ∆R calculation program, *deltar*^37^, with the Marine20 calibration curve ^38^ (Fig. 2; Supplemental Data Tables 2, 3 and 4). Δ^14^C was calculated following using the following equation: $\Delta {}^{14}\mathrm{C}=(\mathrm{F }\times {e}^{\lambda t}-1)\times 1000$ where $\lambda =1/8267$ years, based on the Cambridge (5730) half-life and *t* is the age of the sample (years before 1950 AD) (Supplementary Fig. 4; Supplemental Data Tables 2, 3). Fraction modern (F) is converted into a radiocarbon age using the Libby half-life of 5568 years^57^ in the following equation:

$${\text{Radiocarbon}}\,{\text{age}} = - 8033 \times {\text{ln}}(F_{m} )$$

### Exclusion of records

The ∆R time series presented for coral 64344 is continuous and complete. However, the ∆R time series presented for 35104 have periods where ∆R has been omitted due to extremely slow coral growth and large uncertainties in ∆R calculations. Coral 35104 exhibits an extreme reduction in growth rate across the interval 2019 to 578 cal BP^28^. Growth rapidly slows from ~ 35 µm/year to ~ 1 µm/year beginning ca. 2000 cal BP. Growth rate is suppressed until ~ 1000 cal BP, when it slowly starts to rise back to ~ 10 µm/year until the coral’s death ~ 600 cal BP. Such a rapid decline in growth rate has not been seen in other black coral growth studies^14,58–60^. Hitt et al.^28^ speculate the change in growth may result from some traumatic event or animal grazing on the coral skeleton.

In this slow-growth region, the evenly spaced U-Th ages become more separated in time. This means that U-Th ages have to be intensively interpolated to match the sampling resolution of ^14^C dates, introducing a large uncertainty. ∆R values in this region are therefore highly reliant on a very low number of U-Th ages (n = 12 over 2019–578 cal BP years vs. n = 32 over 2982–2019 cal BP). This issue is exacerbated by elevated detrital Th during the slow-growth interval where the initial ^230/232^Th values of these ages (54 ± 11) are notably higher than the older portion of this coral (14 ± 7), and for other corals in the Southwest Pacific (1–40 for other corals studied here as well as those in Komugabe-Dixson et al.^14^). These high initial ^230/232^Th values result in U-Th age uncertainties up to ten times larger between 2019 and 574 cal BP compared with 2982–2019 cal BP. Together these factors expand the error in ∆R such that measurements are not statistically distinguishable across the 2019 to 578 cal BP interval (Supplementary Fig. 5).

To illustrate: calculating ∆R across the entire 35104 record using the full suite of the U-Th dates shows a multi-centennial ∆R cycle with an amplitude of ~ 500 ^14^Cyr (Suppementary Figs. 5, 6). This contradicts ∆R reconstructions over this interval from the other existing New Zealand black coral (NIWA 64344) and black coral ∆R records from the South Tasman Sea^14^. There is no statistically significant relationship between the New Zealand ∆R records where they overlap as the large multi-centennial ∆R cycle observed in NIWA 35104 is not observed in NIWA 64344. The South Tasman Sea corals do show a more negative ∆R values over 2000–1000 cal BP interval; however, it is significantly less than NIWA 35,104 (about 250 ^14^Cyrs vs. the 400–600 ^14^Cyrs in NIWA 35,104). The South Tasman Sea corals also show an opposite change than NIWA 35104, where ∆R shifts from more negative ∆R to more positive ∆R values from 2000 to 1000 cal BP that occurs over many centuries. The shapes of these changes are also very different, where the South Tasma Sea Corals show a gradual ∆R trend and NIWA 35104 shows a complete cycle.

We therefore conclude there is significant risk in trying to interpret the 2019 to 578 cal BP section of 35104 ∆R record with the current U-Th age datasets. We therefore remove the 35104 ∆R data over the 2019 to 578 cal BP interval from all figures, calculations and data interpretations in the main text but include the data in the supplementary file for completeness.

## Supplementary Information

Supplementary Information 1.

Supplementary Information 2.

## Data Availability

Data used in this work is available in the supplementary data file provided.
