# Supplementary material for: Natural cycles in South Pacific Gyre strength and the Southern Annular Mode
Source: Sci Rep. 2022 Oct 27;12:18090. doi: 10.1038/s41598-022-22184-2 (PMC9613651; doi:10.1038/s41598-022-22184-2)
Supplement: Supplementary file 1 — Supplementary Information 1. [file 41598_2022_22184_MOESM1_ESM.docx]

**Supplementary Info For:**

**Natural Cycles in South Pacific Gyre Strength and the Southern Annular Mode**

Nicholas T. Hitt^1,2*^, Daniel J. Sinclair^1^, Helen L. Neil^2^, Stewart J. Fallon^3^, Aimée Komugabe-Dixson^3,4^, Denise Fernandez^2^, Philip J. Sutton^2^, John C. Hellstrom^5^

^1^Victoria University of Wellington, Wellington, New Zealand

^2^National Institute of Water and Atmospheric Research, Wellington, New Zealand

^3^Australian National University, Canberra, Australia

^4^Global Fishing Watch, Washington DC, USA

^5^University of Melbourne, Melbourne, Australia

*Corresponding author: Nicholas Hitt at Nicholas.hitt@vuw.ac.nz

**DESCRIPTION:**

This file contains all supplementary text, figures, and tables for the accompanying manuscript.

Supplemental Text:

Discussion of Black Coral ∆^14^C and Paleo-SAM Relationship

Marine20 for the first 12,000 years is based on atmospheric northern hemisphere data and reconstructed changes in CO_2_ constrained within a global ocean/atmosphere/biosphere carbon cycle model^3^. This may create some uncertainty when determining if black coral ∆R variations are indeed forced by climatological changes from the Southern Annular Mode, or just some function of poor constraints and modelling techniques within Marine20. We ensure black coral radiocarbon is indeed climatologically forced by paleo-variations in the SAM by providing a granular investigation into black coral ∆^14^C below following the methods used in Komugabe-Dixson et al., ^5^.

Komugabe-Dixson et al., ^5^ illustrated that black coral ∆^14^C reflected changes in ocean circulation by examining the offset (i.e. the difference) between black coral ∆^14^C, and Marine13 and SHCal13. They showed that there was up to a positive 20‰ shift between the corals and Marine13 during periods with stronger subtropical circulation. This shift reflects a change towards the movement of subtropical waters that are more ventilated to the atmosphere and have a greater CO_2_ air-sea gas exchange.

We undertake a similar analysis, where we find the residual difference between coral ∆^14^C and SHCal20^4^ and then calculate z-scores of the residual ∆^14^C values for comparison against the Abram et al., ^6^ Paleo-SAM record (Supp. Fig. 9). The residual difference here attempts to show that variations in the residual difference between black coral ∆^14^C and SHCal20^4^ is indeed a result of ocean circulation and suggests the similar ∆R variations are not a function of poor constraints and modelling techniques within Marine20.

The z-scored residual difference between black coral ∆^14^C and the SHCal20^4^ calibration curve shows a good positive relationship with the Abram et al.,^6^ Paleo-SAM index, where more negative SAM periods coincide with a negative residual difference in black coral ∆^14^C. Conversely periods with a more positive SAM period show a positive residual difference in black coral ∆^14^C. Although these differences are comparatively small (<5‰) to those residual differences observed in Komugabe-Dixson et al., ^5^, they are nevertheless significant which shows that our reconstruction of black coral radiocarbon is climatically driven.

The correlation between the black coral z-score normalized residual difference in ∆^14^C and the Abram et al., 2014 Paleo-SAM index is slightly weaker in absolutely magnitude than the correlation between black coral R and ∆R and the Paleo-SAM index (r = 0.37, p < 0.05 (∆^14^C) vs. r = -0.49 to -0.54, p < 0.001 (R & ∆R)), however all three correlations are significant. The slightly weaker correlation could be interpreted that the calibration curves used to derive R and ∆R values do have some, albeit very minor, influence on the relationship between R and ∆R and the Paleo-SAM index.

The above analysis only compares the residual difference between black coral and SHCal20 ∆^14^C against the paleo-SAM index over the last 950 cal BP. Therefore, to ensure that variations in black coral radiocarbon do indeed reflect climate variability over the last 3000 years and the correlation between radiocarbon and climate are not a feature over the last millennium, we compare the offset between black coral ∆R with the residual difference between black coral and SHCal20 ∆^14^C in both corals (64344 and 35104; Supp. Fig 11). We find that ∆R has a strong negative relationship with the residual difference between black coral and SHCal20 ∆^14^C in both corals. This shows that variability in ∆R over the last 3000 years co-varies with the residual difference in black coral ∆^14^C and the SHCal20 ∆^14^C which has been established to reflect ocean circulation^5^.

We therefore conclude the relationship between black coral radiocarbon in the East Auckland Current over the late Holocene does appear to reflect Paleo-SAM conditions/climatic variability regardless of the metric used (∆^14^C, R, or ∆R) and propose that any influence from the calibration curves used to produce R and ∆R is likely minor.

Supplemental Table 1: A collection of black coral information. Black coral location, species, depth, lifespan, the number of radiocarbon and U/Th dates and mean ∆R. U-Th ages are corrected for initial thorium following protocols in Hellstrom,^1^. Weighted mean ∆R is calculated according to Bevington^2^ and the uncertainty is the standard deviation of ∆R scaled by the square root of the number of observations. All uncertainties are 1σ.

**
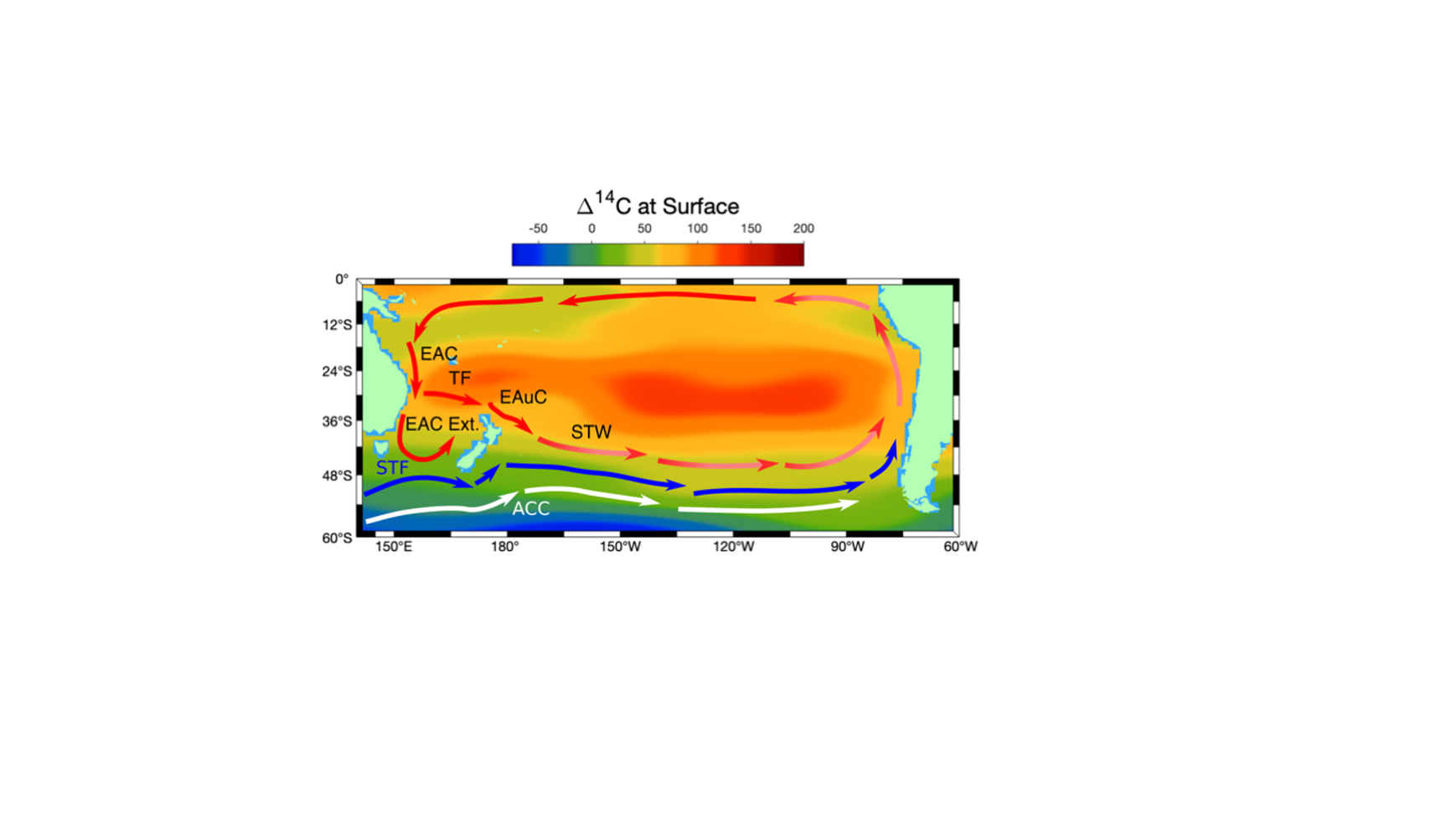
**

Supp Fig. 1: South Pacific gridded modern surface ∆^14^C data. A schematic of South Pacific Gyre Ocean currents around New Zealand overlaid on gridded modern surface ∆^14^C data (GLODAP). EAC – East Australian Current; TF – Tasman Front; EAC Ext. – East Australian Current Extension; STF – Subtropical Front; EAuC – East Auckland Current; STW – Subtropical Water; ACC – Antarctic Circumpolar Current. Red arrows indicate warm subtropical currents, blue arrows indicate cool subtropical currents and white arrows indicate polar currents. Data here represents global gridded surface ∆^14^C data at present. The dynamic range of pre-bomb ∆^14^C may be less than show here; however, the same patterns in ∆^14^C would exist.


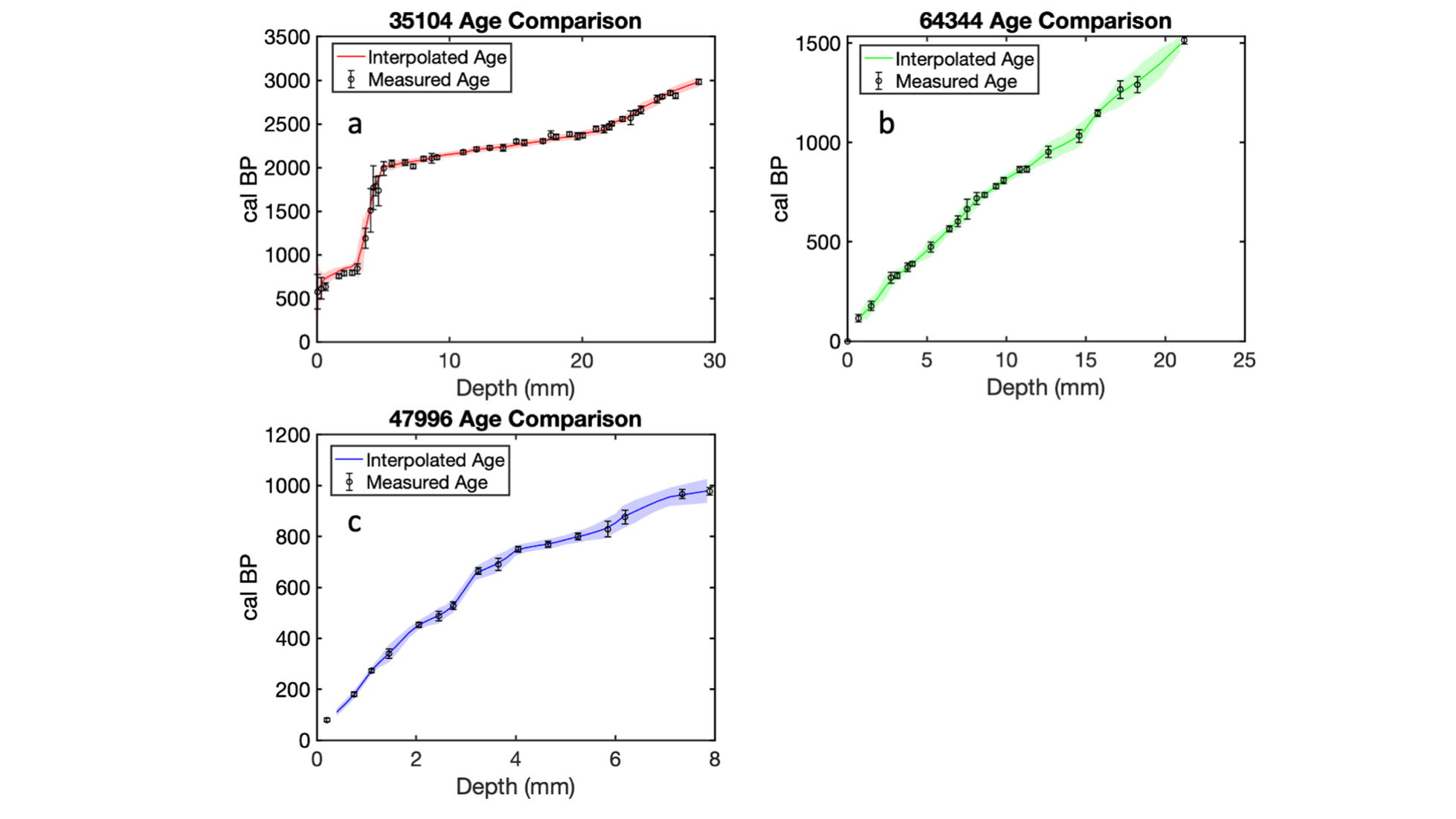


Supp. Fig. 2: Interpolated and initial Th corrected age models. A comparison between initial Th corrected ages (symbols) and interpolated age models from COPRA (colored lines). All uncertainties shown are 2σ. Depth progresses from the outer coral (0mm) to inner coral (e.g. 20mm).


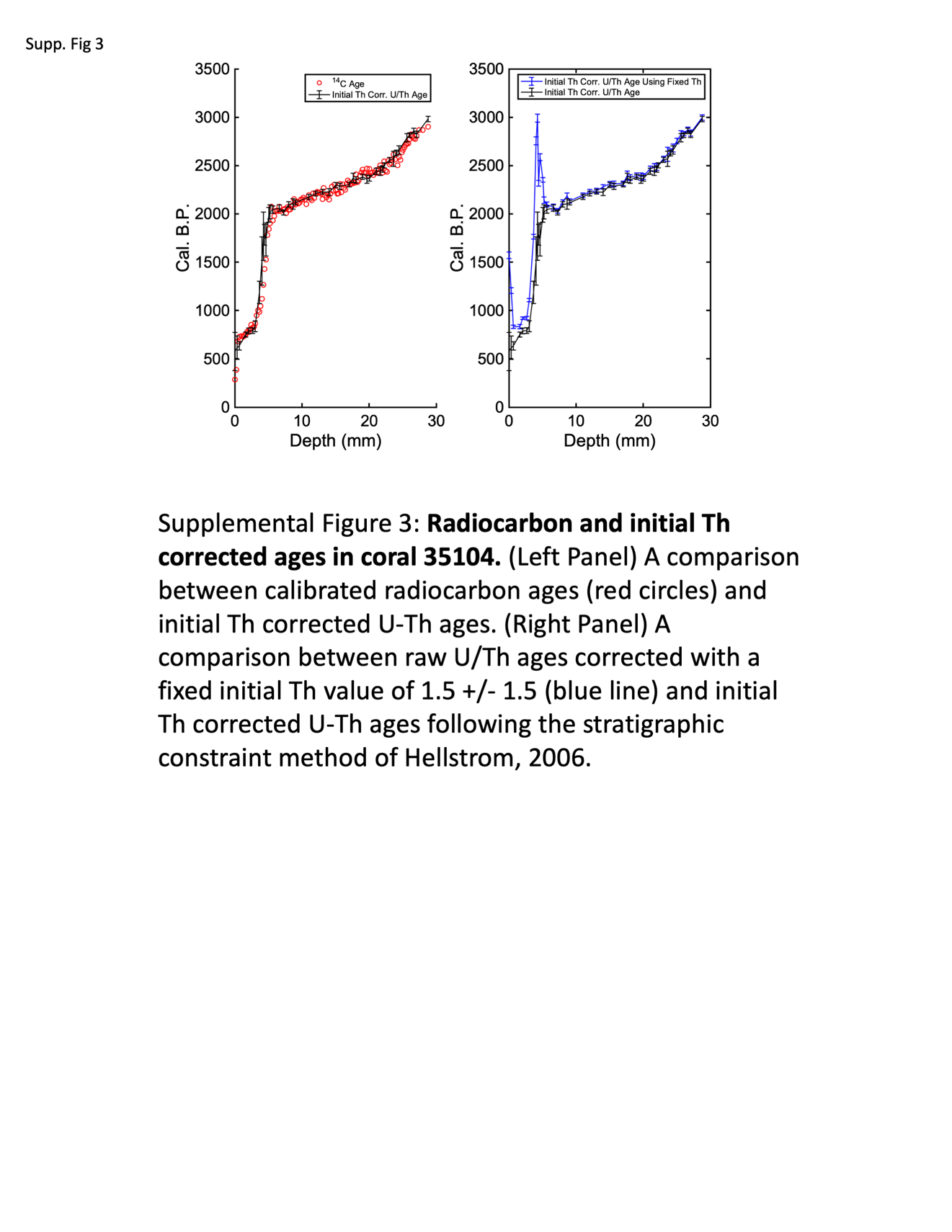


Supp. Fig. 3: Radiocarbon and initial Th corrected models in coral 35104. (Left Panel) A comparison between calibrated radiocarbon ages (red circles) and initial Th corrected U-Th ages. (Right Panel) A comparison between raw U/Th ages corrected with a fixed initial Th value of 1.5 +/- 1.5 (blue line) and initial Th corrected U-Th ages following the stratigraphic constraint method of Hellstrom ^1^.


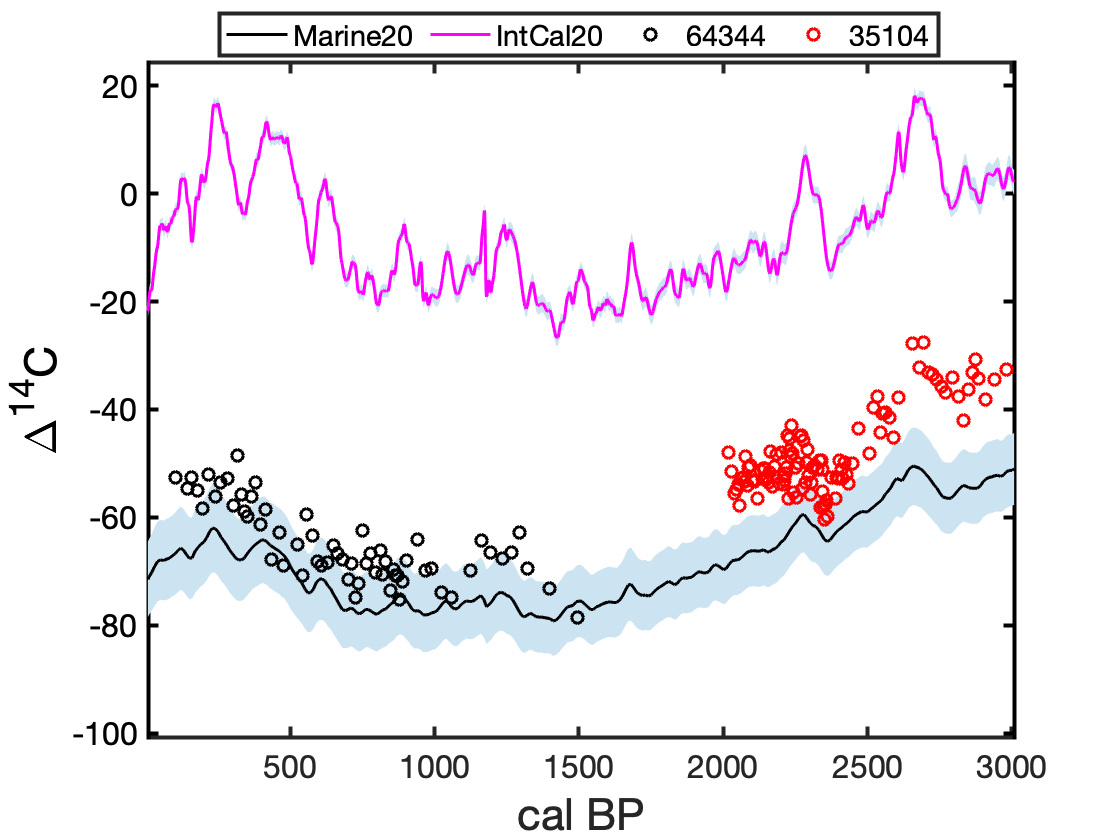


Supp. Fig. 4: Coral, IntCal20, and Marine20 ∆^14^C. Coral ∆^14^C plotted over ∆^14^C from Marine20^3^ and Intcal20 ^8^. Uncertainties shown are 1σ.

**
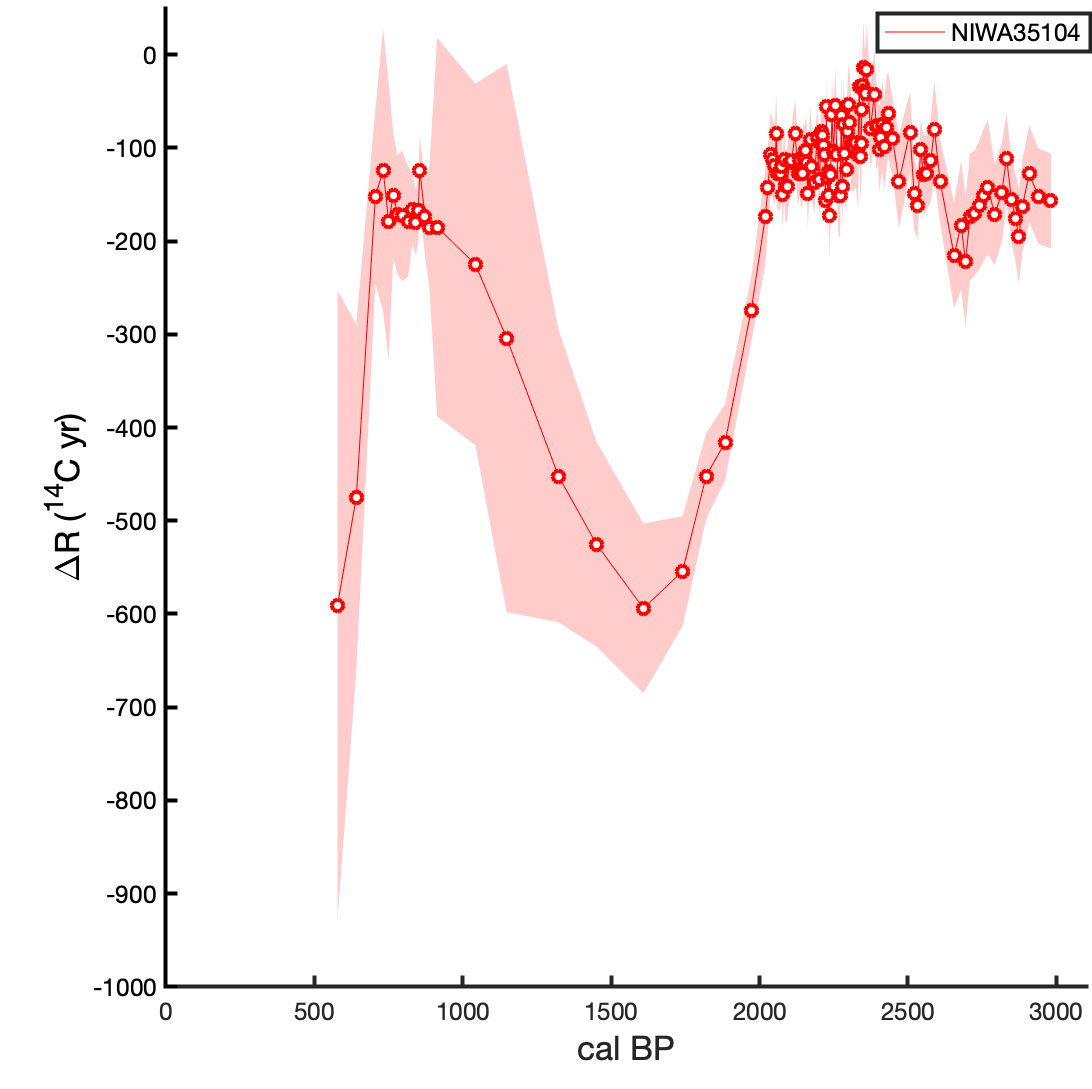
**

Supp. Fig. 5: The Complete Coral 35104 ∆R Record. ∆R in coral 35104 from 2982 to 578 cal BP. ∆R shows an unrealistic oscillation with an amplitude of ~500 ^14^Cyr over the 2019 to 578 cal BP interval. This interval corresponds to unusually slow growth and high detrital Th values (Supp. Fig. 3). The unusually slow growth comprises accurately interpolating U-Th age to the frequency of ^14^C dates, which contributes to the large ∆R oscillations. The high detrital Th values affect U-Th date precision which contributes to ∆R that are not significantly different from one another over this period.

**
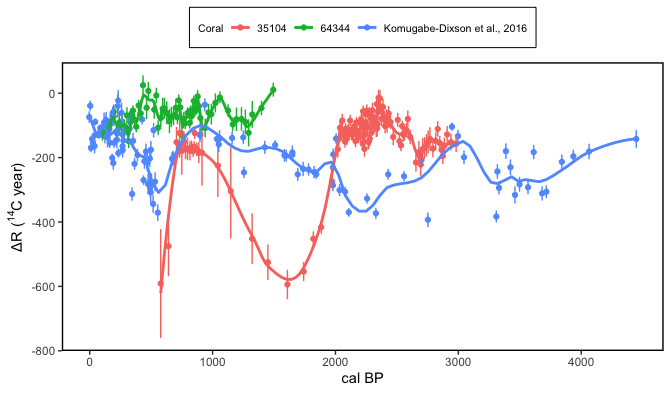
**

Supp. Fig. 6: All New Zealand and South Tasman Sea ∆R. ∆R in coral 35104 (red dots), coral 64344 (green dots), and the South Tasman Sea from Komugabe-Dixson et al.,^5^ (blue dots). Colored lines show a loess-filtered fit to the data presented to indicate trends in ∆R. Uncertainties are shown by the error bars and are 1σ.


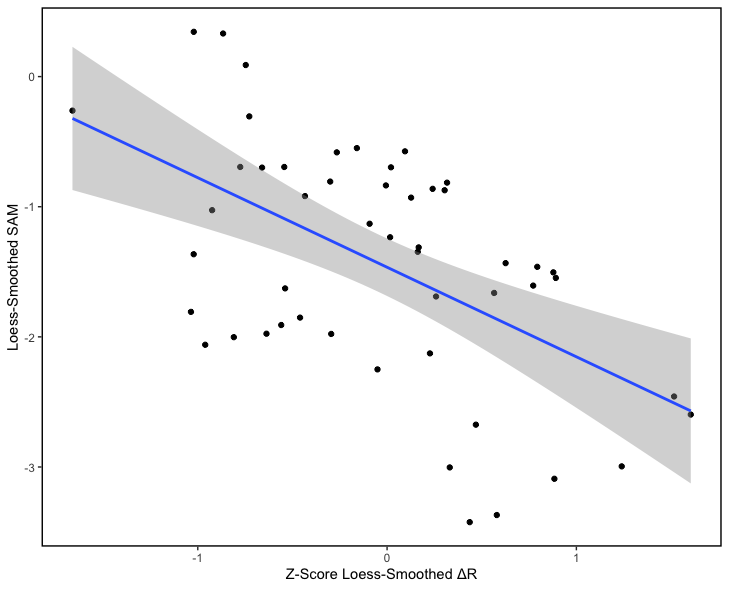


Supp. Fig. 7: ∆R Z-Scores vs. SAM Regression. Loess-filtered coral ∆R z-scores plotted against Loess-filtered paleo-SAM data from Abram, et al. ^5^. The linear regression is calculated according to the protocols in York, et al. ^6^, which account for uncertainty in both variables. Uncertainty bounds are 2σ and are shown with the shaded areas. P < 0.0001; R^2^ = 0.29. The regression equation is:

$$y_{SAM}= -0.6881\left( x_{\Delta R} \right)-1.4652$$

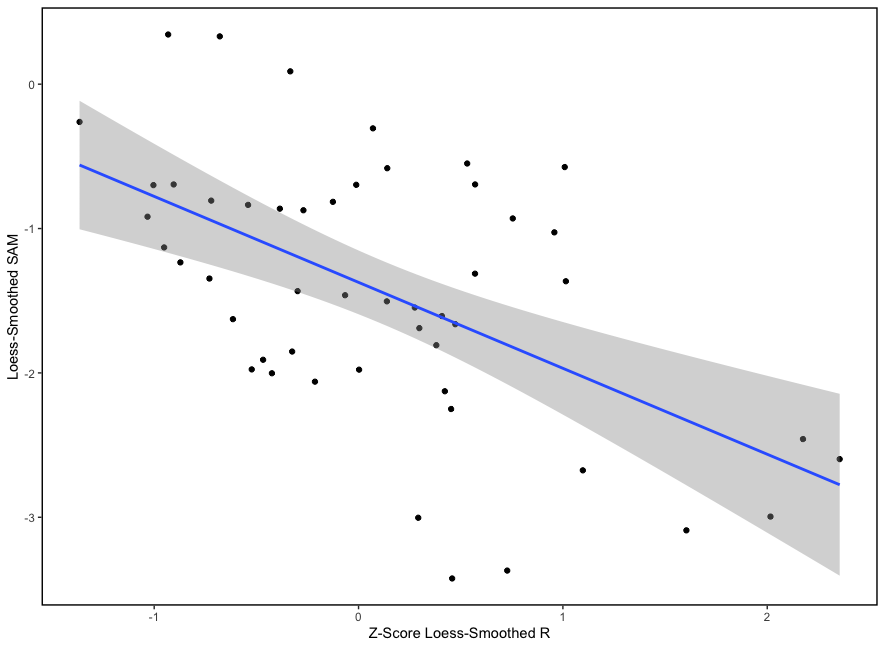


Supp. Fig. 8: R Z-Scores vs. SAM Regression. Loess-filtered coral R z-scores plotted against Loess-filtered paleo-SAM data from Abram, et al. ^6^. The linear regression is calculated according to the protocols in York, et al. ^7^, which account for uncertainty in both variables. Uncertainty bounds are 2σ and are shown with the shaded areas. P < 0.0001; R^2^ = 0.30. The regression equation is:

$$y_{SAM}= -0.59\left( x_{R} \right)-1.3728$$

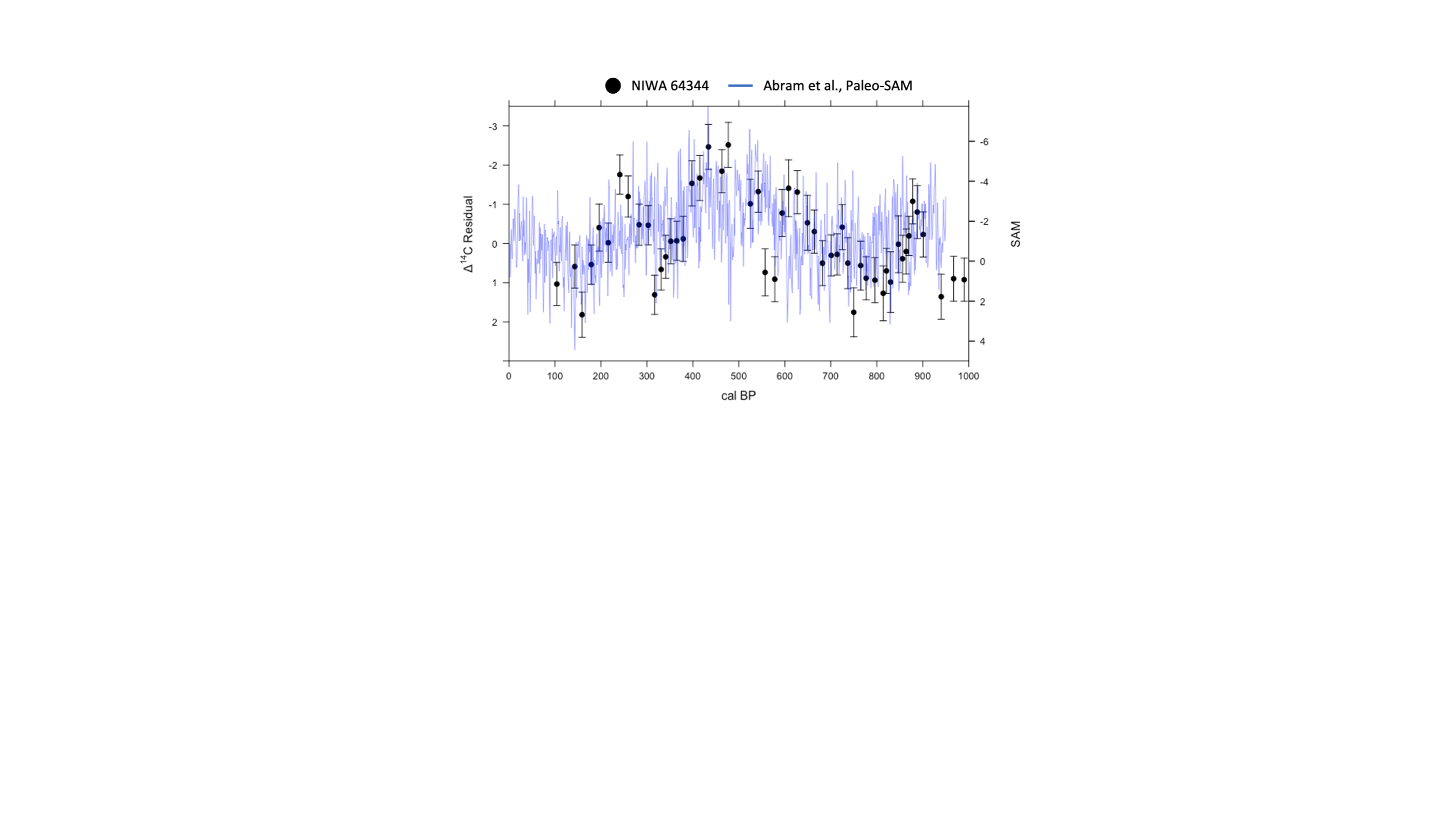


Supp. Fig. 9: ∆^14^C Z-Scores vs. SAM Over the Last 1000 Years. ∆R in NIWA 64344 (black dots) and the Abram et al., ^6^ Paleo-SAM index over the last 1000 years (blue line).


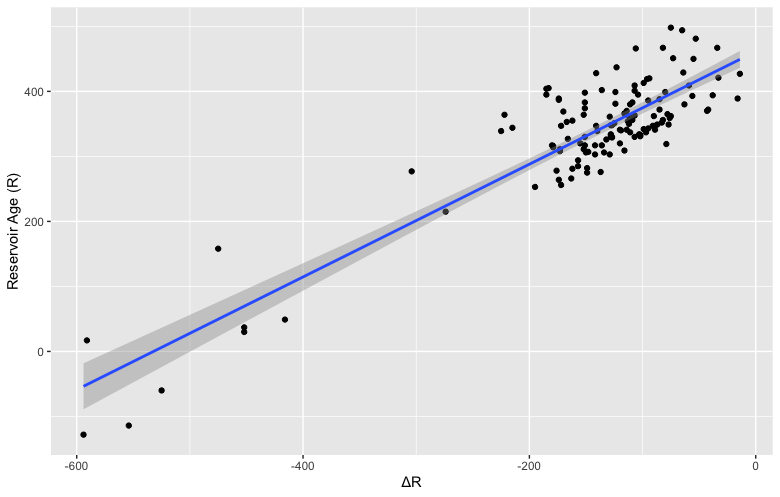


Supp. Fig. 10: Black Coral ∆R vs. R Regression. A linear regression of black coral ∆R vs. R. The linear regression is calculated according to the protocols in York, et al. ^7^, which account for uncertainty in both variables. Uncertainty bounds are 2σ and are shown with the shaded areas. P < 0.0001; R^2^ = 0.78. The regression equation is:

$$R= 0.805\left( x_{\Delta R} \right)+448.9462$$

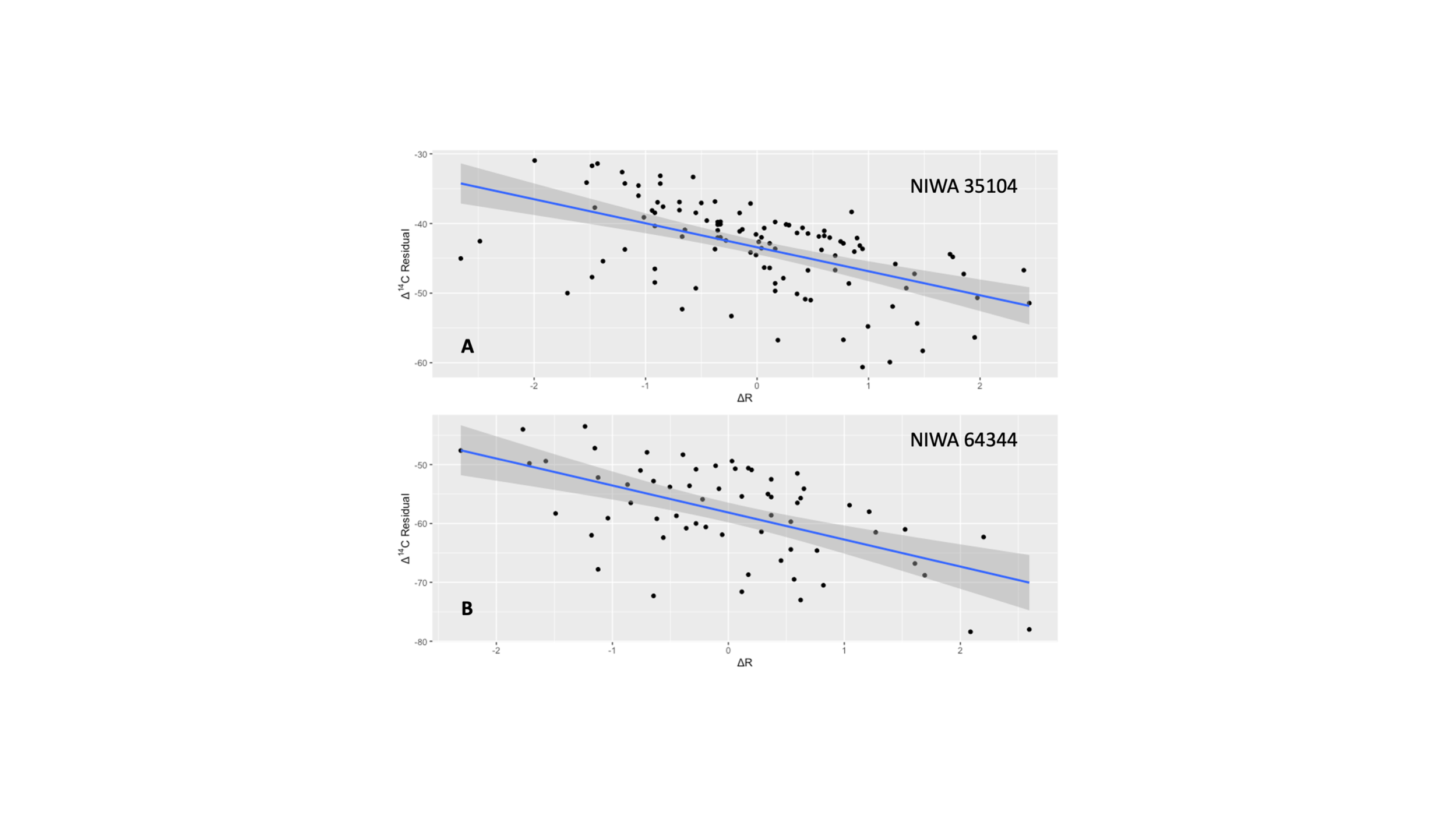


Supp. Fig. 11: Black Coral ∆R vs. ∆^14^C Residual Regression. A linear regression of black coral ∆R vs. the residual difference in black coral ∆^14^C and SHCal20 ∆^14^C. Linear regressions are calculated according to the protocols in York, et al. ^6^, which account for uncertainty in both variables. Uncertainty bounds are 2σ and are shown with the shaded areas. P < 0.0001; R^2^ = 0.99 for both linear regressions.

The regression equation for NIWA 35104 (Panel A) is:

$$\Delta^{14}C Residual= -0.11\left( x_{\Delta R} \right)-3.1$$

The regression equation for NIWA 64344 (Panel B) is:

$$\Delta^{14}C Residual= -0.13\left( x_{\Delta R} \right)-6.8$$

**REFERENCES:**

S1 Hellstrom, J. U-Th dating of speleothems with high initial 230Th using stratigraphical constraint. *Quaternary Geochronology* **1**, 289-295, doi:10.1016/j.quageo.2007.01.004 (2006).

S2 Bevington, P. R., Robinson, D. K., Blair, J. M., Mallinckrodt, A. J., & McKay, S. (1993). Data reduction and error analysis for the physical sciences. Computers in Physics, 7(4), 415-416.

S3 Heaton, T. J. *et al.* MARINE20—THE MARINE RADIOCARBON AGE CALIBRATION CURVE (0–55,000 CAL BP). *Radiocarbon*, 1-42, doi:10.1017/RDC.2020.68 (2020).

S4 Hogg, A., Heaton, T., Hua, Q., Palmer, J., Turney, C., Southon, J., . . . Wacker, L. (2020). SHCal20 Southern Hemisphere Calibration, 0–55,000 Years cal BP. Radiocarbon, 62(4), 759-778. doi:10.1017/RDC.2020.59

S5 Komugabe-Dixson, A. F., Fallon, S. J., Eggins, S. M. & Thresher, R. E. Radiocarbon evidence for mid-late Holocene changes in southwest Pacific Ocean circulation. *Paleoceanography* **31**, 971-985, doi:10.1002/2016PA002929 (2016).

S6 Abram, N. J. *et al.* Evolution of the Southern Annular Mode during the past millennium. *Nat. Clim. Change* **4**, 564–569 (2014).

S7 Derek York, Norman M. Evensen, Margarita López Martı́nez & Jonás De Basabe Delgado. Unified equations for the slope, intercept, and standard errors of the best straight line. *Am. J. Phys.* **72**, 367–375 (2004).

S8 Reimer, P., Austin, W., Bard, E., Bayliss, A., Blackwell, P., Bronk Ramsey, C., . . . Talamo, S. (2020). The IntCal20 Northern Hemisphere Radiocarbon Age Calibration Curve (0–55 cal kBP). Radiocarbon, 62(4), 725-757. doi:10.1017/RDC.2020.41
